# The Treatment of Arteriosclerosis

**Published:** 1906-02-10

**Authors:** 


### THE TREATMENT OF ARTERIO-SCLEROSIS.

In a lecture on this subject Sir James Barr [1] first reviews the question of etiology with a view to define the measures necessary to prevent the development of arterial degeneration. In this respect he deals with the influence of nitrogenous food, alcohol, syphilis, lead, enteric fever, and other agents, and suggests that the toxin of the *Bacillus coli* may have an important influence in the production of arterio-sclerosis. In dealing with the established disease diet has the first place. Chittenden has clearly demonstrated that all classes of men can not only live, but can enjoy perfect health and strength, both mental and physical, on one-half or one-third of the proteid food described as necessary in the standard dietaries of Voit and others. Undoubtedly it is excess of nitrogenous materials in the modern dietary scheme which is responsible for much of the arterio-sclerosis so frequently met with in practice. Slow eating and thoroughness in mastication are other factors which by indirect methods tend to delay the advance of arterio-sclerosis. Outdoor exercise falls into the same category, but the dangers of excess must not be forgotten; danger also attaches to mountain climbing and even ascents to high altitudes by means of mountain railways. The baths of Harrogate, Llandrindod Wells, Buxton, Bath, Aix-les-Bains, Vichy, etc., often do much good in the treatment of these cases. In regard to drugs it is to be noted that the thyroid preparations are extremely useful in arterio-sclerosis. They both dilate the arteries and increase tissue metabolism; the dose must, of course, not be pushed so as to produce the symptoms of thyroidism. Iodine is often even more valuable; it acts by stimulating the thyroid gland. It may be prescribed in the form of tincture, or as a syrup with tannic acid, or the iodides may be ordered. Adrenalin as an agent which raises blood pressure would seem to be contra-indicated in cases of arterio-sclerosis, but when combined with thyroid or iodine it may do good by maintaining arterial tone and stimulating the heart. The benzoates are of great use, especially where the kidneys are involved, and recently the hippurates have been introduced by Dr. George Oliver, and have proved of value for lowering the blood pressure. The nitrites are valuable for emergencies, but their action is too evanescent for a sustained effect. Indirectly, that is by clearing out the intestinal canal and depleting the intestinal vessels, sodium sulphate and other saline purgatives are also of great importance in the treatment of cases of arterio-sclerosis.

[1] Brit. Med. Jour., January 20, 1906.

